# Incorporating Cystatin C to Predict Methotrexate Elimination in Patients with CNS Lymphoma and Suspicious Renal Function

**DOI:** 10.1155/2018/7169897

**Published:** 2018-03-26

**Authors:** Jason N. Barreto, Allison L. McClanahan, Andrew D. Rule, Carrie A. Thompson, Erin Frazee

**Affiliations:** ^1^Department of Pharmacy, Mayo Clinic, Rochester, MN, USA; ^2^Division of Hematology, Department of Internal Medicine, Mayo Clinic, Rochester, MN, USA; ^3^Division of Nephrology and Hypertension, Department of Internal Medicine, Mayo Clinic, Rochester, MN, USA; ^4^Division of Epidemiology, Department of Health Sciences Research, Mayo Clinic, Rochester, MN, USA; ^5^Robert D. and Patricia E. Kern Center for the Science of Health Care Delivery, Mayo Clinic, Rochester, MN, USA

## Abstract

High-dose methotrexate (MTX; ≥1 g/m^2^) is a renally eliminated and nephrotoxic first-line therapy for central nervous system (CNS) lymphoma. Creatinine-based estimation of renal function is the recommended approach to dosing MTX in these cases, but nonrenal determinants of creatinine production and elimination in cancer patients such as malnutrition and cachexia lead to overestimation of glomerular filtration rate (GFR) by this method and a heightened risk for drug toxicity. Serum cystatin C is one of the first readily available, relatively inexpensive, endogenous biomarkers to emerge as a practical adjunct to creatinine for estimation of renal function for drug dosing. In this report, we describe two cases where cystatin C was used in conjunction with creatinine to inform MTX dosing for CNS lymphoma. In both cases, the estimated GFR was nearly 40% lower with the combination of the two biomarkers compared to creatinine-only estimates. Empiric MTX dose reductions as a product of these results likely spared the patients sustained exposure to toxic drug concentrations and facilitated earlier administration of supportive care interventions. Further prospective investigations with validated dosing regimens including cystatin C are warranted for high-dose MTX.

## 1. Introduction

High-dose methotrexate (MTX) is currently the treatment of choice for central nervous system (CNS) lymphoma [1]. Although the dose for MTX has ranged in previous studies from 3.5–12 g/m^2^, doses in excess of 1 g/m^2^ have been shown to achieve sufficient CNS penetration for prophylaxis and treatment of lymphoma [2]. MTX is a renally eliminated, nephrotoxic antineoplastic agent that requires an accurate assessment of preexisting kidney function as determined by glomerular filtration rate (GFR) for optimal dosing [3]. Use of high-dose MTX in patients with unrecognized renal dysfunction could lead to acute kidney injury (AKI), increased risk of systemic toxicity from prolonged drug exposure, and an extended hospitalization due to prolonged MTX clearance [4]. Exposure to exceedingly high concentrations of MTX for a protracted period of time is known to cause dermatitis, hepatitis, debilitating mucositis, and life-threatening myelosuppression [4, 5].

A serum creatinine-based estimation of GFR calculated using the Cockcroft-Gault (CG) formula is the recommended equation to estimate renal function when determining the MTX dose for a patient with CNS lymphoma [6, 7]. Measured creatinine clearance with a timed urine collection may also be used, but it is cumbersome, time consuming, and may be inaccurate in patients without an indwelling urinary catheter. Creatinine production is often reduced in patients with malignancies due to reduced skeletal muscle mass (the source of creatinine generation), and acute or chronic deconditioning, which can lead to overestimation of GFR with serum creatinine [8]. Cystatin C is one of the first endogenous GFR markers to emerge as a practical alternative to creatinine in the last several decades. It is much less influenced by muscle mass than creatinine, though it does have different nonrenal confounders. Its use has been widely validated across the continuum of care, and evidence indicates that the combined use of serum creatinine and cystatin C to estimate GFR more accurately predicts true renal function than use of either marker alone [9].

It is unclear whether use of cystatin C combined with serum creatinine to estimate GFR at the time of MTX dosing could more accurately predict MTX concentrations than a standard creatinine-only approach. More complete characterization of a patient's renal profile surrounding MTX administration could be the first step to identifying methods to optimize both efficacy and safety of this drug for CNS lymphoma and other malignancies. Herein, we present two cases with CNS lymphoma where the Chronic Kidney Disease Epidemiology Collaborative equation with both serum creatinine and cystatin C (CKD EPI_Cr-CysC_) was used for GFR estimation at the time of MTX dosing and throughout the therapy.

## 2. Case Presentation

### 2.1. Case 1

A 51-year-old (172 cm, 72.7 kg) man with a history of diffuse large B-cell lymphoma (DLBCL) presented with acute neurologic decline including a witnessed generalized tonic-clonic seizure prompting hospital admission. The patient underwent an extensive workup encompassing 28 days of persistent functional decline and progressive paralysis. The investigation concluded with a sural nerve biopsy that revealed neurolymphomatosis (DLBCL) with secondary nerve microvasculitis.

The decision was made to treat with 8 grams/m^2^ and administer 15 grams of MTX based on a CG estimated creatinine clearance of >120 mL/min calculated using a serum creatinine of <0.4 mg/dL (comparable to available creatinine values in 1 month prior to admission) and body surface area (BSA) of 1.88 m^2^. Unfortunately, dysautonomia associated with the neurologic syndrome precipitated a hypotensive event requiring vasopressor support and a delay of chemotherapy. This acute decompensation prompted increased scrutiny of the patient's renal function beyond standard serum creatinine measurements. A cystatin C measurement returned at 1.81 mg/L, which indicated a CKD EPI_Cr-CysC_ eGFR of 69 mL/min/1.73 m^2^ (70 mL/min). The MTX dose was subsequently decreased by 20% to 12 grams in accordance with the impaired renal function. MTX was administered followed by leucovorin rescue according to protocol [7]. Routine monitoring included daily serum creatinine and cystatin C concentrations, urine output, and serum MTX concentrations 48 hours after the infusion initiation and every 24 hours thereafter. On day +2 of MTX therapy, serum creatinine remained 0.4 mg/dL, cystatin C was 1.86 mg/L (CKD EPI_Cr-CysC_ eGFR of 68 mL/min/1.73 m^2^, 74 mL/min), and the corresponding serum MTX level was 0.44 *μ*mol/L (desired level < 0.1 *μ*mol/L). Daily serum MTX concentrations are shown in Table 1. During this timeframe, the patient experienced mild hypokalemia, grade 1 elevation in aspartate aminotransferase without clinically evident symptoms. MTX concentrations were measured until a level of <0.1 *μ*mol/L was achieved at day +6 at which point supportive care measures (leucovorin, intravenous fluids, sodium bicarbonate, and furosemide) were discontinued. Continued observation over the next 7 days demonstrated no change in the patient's neurologic status, and rituximab (375 mg/m^2^) in combination with 5 days of high-dose methylprednisolone (1 gram) were administered. Unfortunately, the patient still showed no improvement over the next several days. The decision was made to pursue comfort measures, and the patient expired soon after.

### 2.2. Case 2

A 38-year-old (177 cm, 78.6 kg) man with a history of relapsed Burkitt's lymphoma with neurolymphomatosis, positive CSF cytology, and likely parenchymal disease based on imaging was admitted for high-dose MTX 8 grams/m^2^ as part of combination chemotherapy. During the previous admission for cycle-1 day 1, 15.8 grams of MTX was administered based on a CG estimated creatinine clearance of >90 mL/min calculated using a serum creatinine of 1.2 mg/dL and BSA of 1.97 m^2^. The 48-hour MTX concentration from this first dose in the cycle was elevated at 5.1 *μ*mol/L, which resulted in a prolonged hospital length of stay until day +8 when the level had fallen to less than 0.1 *μ*mol/L. With the exception of severe nausea and vomiting, the patient experienced no clinical or laboratory abnormalities. By day +8, his nausea and vomiting had resolved, and the patient was discharged.

Evaluation prior to dose 2 (cycle 1, day 15) revealed no appreciable changes to body habitus or serum creatinine concentration. The recommended dose would have again been 15.8 grams of MTX, but given the previous elevations in serum MTX concentrations, an empiric 20% dose decrease was planned and a cystatin C was checked. The serum cystatin C measurement prior to dose 2 was 1.99 mg/L corresponding to a CKD EPI_Cr-CysC_ estimated GFR of 47 mL/min/1.73 m^2^ (54 mL/min). Based on this information, the MTX dose was reduced an additional 30% to 9 grams and leucovorin rescue was preemptively increased from the standard 25 mg to 100 mg every 6 hours and ordered to start 24 hours after the MTX infusion as per protocol.

As with Case 1, routine monitoring included daily serum creatinine and cystatin C concentrations, urine output, a serum MTX concentration at day +2 and as clinically indicated thereafter. On day +2 of MTX therapy (cycle 1 day 17), serum creatinine increased to 1.6 mg/dL from 1.1 mg/dL while cystatin C remained steady at 1.85 mg/L and the serum MTX level returned at 1.7 *μ*mol/L. Throughout treatment with dose 2, the patient never experienced any adverse clinical effects. By day +5, the MTX concentration declined to the goal level of less than 0.1 *μ*mol/L, and the patient was discharged without signs and symptoms of any toxicity. Unfortunately, shortly after this discharge, the patient returned to an outpatient visit with debilitating leg pain and a white blood cell count of 52,400 cells/mm^3^ and was confirmed to have disease progression. Systemic combination chemotherapy was changed to rituximab, gemcitabine, vinorelbine, and prednisone.

## 3. Discussion

These two cases highlight the potential utility of cystatin C to assist with drug dosing in patients with lymphoma receiving MTX. In both of these circumstances, the serum creatinine and corresponding estimated CG creatinine clearance overestimated the patients' GFR. In the second case, an overestimated creatinine-based GFR led to a larger MTX dose than what was appropriate, resulting in a prolonged hospital stay. In both cases, cystatin C incorporated into the GFR estimation predicted more acceptable serum concentrations of MTX.

An accurate assessment of renal function is critical at the time of MTX initiation to ensure that the selected dose optimizes tumor kill without subjecting the host to unnecessary toxicity [10]. Although direct GFR measurement with an exogenous agent such as iothalamate would be ideal, application of this method to all patients receiving nephrotoxic pharmacotherapies is time intensive, resource demanding, and impractical in most acute care settings [11]. Even approximation of GFR with a 24 h urinary creatinine clearance is challenging due to urine collection errors, extended intervals of time needed for the collection, and delayed turnaround for results [12]. Therefore, the current recommended tool to estimate drug elimination at the bedside is serum creatinine [13]. However, there are many nonrenal factors that contribute to altered serum creatinine production and elimination, particularly in patients with malignancy, which can lead to a greater risk for an inaccurate renal estimate [8]. Cystatin C is an endogenous low-molecular weight protein produced at a constant rate by all nucleated cells. Unlike creatinine which undergoes proximal tubular secretion in addition to filtration thus overestimating GFR, cystatin C is freely filtered in the glomerulus and >99% is reabsorbed and catabolized in proximal tubular cells [14]. Non-GFR determinants of cystatin C and creatinine differ, with the former being less influenced by body composition and nutritional status, but more affected by inflammation and cell turnover [15–17]. Cystatin C distribution is confined to the extracellular space rather than the more broadly distributed serum creatinine. This volume of distribution difference confers a kinetic advantage to cystatin C and suggests that it could more rapidly respond to changes in underlying kidney function than creatinine [18]. As cystatin C is recommended for use in many settings including in the confirmatory diagnosis of chronic kidney disease, it is widely accessible, reasonably priced, has a standardized and validated assay and internet-based publicly available calculators exist to derive a GFR estimate.

Some studies have suggested that cystatin C could serve as a marker for disease activity and prognosis in lymphoma, independent of renal function [19, 20]. However, recent data indicated that cystatin was not associated with overall survival in patients with DLBCL [21]. Furthermore, cystatin C was found to be a useful marker of renal function in pediatric patients receiving methotrexate [22].

Single plasma MTX concentrations which exceed 0.99 *µ*mol/L between 42 and 48 hours after drug administration have been associated with a higher incidence of MTX toxicity [23]. Additionally, the total duration of exposure to MTX concentrations greater 0.1 *µ*mol/L is a primary determinant of consequent bone marrow suppression [5]. Delayed recognition of toxicity is a missed opportunity to institute supportive measures such as increased leucovorin dose to limit the severity of complications [10]. Since the optimal dose of methotrexate remains unknown, it is important to accurately evaluate all pharmacokinetic parameters, including drug clearance, in order to minimize toxicity without compromising efficacy until prospective, randomized trials can compare superiority of one dose to another [2]. In Case 2, the elevated baseline cystatin C, despite a normal creatinine clearance, prompted an MTX dose decrease that was even greater than that originally planned (44% instead of 20%) and a preemptive increase of leucovorin (100 mg every 6 hours instead of 25 mg every 6 hours). Even though the serum MTX concentration was still elevated at 48-hours (1.7 *µ*mol/L, goal < 1.0 *µ*mol/L), this empiric dose adjustment and supportive care strategy were the likely explanation for a more limited degree and duration of exposure in dose 2 relative to dose 1 (5 days versus 8 days, resp.). One could assume that if the dose had only been reduced by 20%, the 48-hour methotrexate concentration would have been much higher than observed, which would have resulted in prolongation of supportive care and an extended hospitalization.

## 4. Conclusion

Serum creatinine-based estimated GFR may overestimate true renal function in patients with lymphoma due to low-serum creatinine concentrations in the setting of reduced skeletal muscle mass and cachexia. Use of serum cystatin C as a method to more comprehensively characterize a patient's GFR to inform MTX dosing represents a novel approach to improve care delivery. Although our cases show promise with this strategy, given that dosing regimens were originally developed using creatinine-based estimates of GFR, prospective investigations of MTX dosing regimens based on cystatin C inclusive GFR estimates are needed. Further studies should seek to clarify to what degree serial cystatin C concentrations would be of utility in such patients.

## Figures and Tables

**Table 1:** Laboratory values for patients longitudinally followed with serum creatinine and cystatin C during methotrexate administration for treatment of CNS lymphoma.

Day	SCr (mg/dL)	CysC (mg/L)	UOP (mL/kg/hr)^a^	eGFR-CG (mL/min)	eGFR CKD EPI_cysC_		eGFR CKD EPI_cr-cysC_		MTX (*µ*mol/L)
					mL/min/1.73 m^2^	mL/min	mL/min/1.73 m^2^	mL/min	
Case 1 (51-year-old male, 172 cm, 72.7 kg, BSA 1.88 m^2^)									
−1	0.4	1.81	5.46	>120	37	40	69	75	—
0	<0.4	—	4.48	>120	—	—	—	—	—
1	<0.4	—	5.36	>120	—	—	—	—	—
2	0.4	1.86	5.41	>120	35	38	68	74	0.44
3	<0.4	1.94	4.79	>120	33	36	66	72	0.18
4	<0.4	1.46	4.30	>120	49	53	81	88	0.16
5	<0.4	1.64	4.81	>120	42	46	75	82	0.13
6	<0.4	—	2.08	>120	—	—	—	—	0.12
7	0.2	1.63	2.15	>120	42	46	86	93	0.07

Case 2 (38-year-old male, 177 cm, 78.6 kg, BSA 1.97 m^2^)									
−1	1.3	1.99	—	87	34	39	47	54	—
0	1.1	1.76	2.99	101	40	46	56	64	—
1	1.6	1.85	2.23	70	38	43	44	50	—
2	1.2	1.92	2.65	93	36	41	50	57	1.7
3	1.1	1.85	4.00	101	38	43	54	61	0.21
4	1.1	1.88	2.99	101	37	42	54	61	0.16
5	—	—	3.26	—	—	—	—	—	0.10

CKD EPI = Chronic Kidney Disease Epidemiology Collaborative; eGFR-CG = estimated GFR calculated from the Cockcroft-Gault equation; CysC = cystatin C; eGFR = estimated glomerular filtration rate; MTX = methotrexate; SCr = serum creatinine; ^a^the daily fluid balance for Case 1 ranged from −5.1 L to +2.1 L and for Case 2 from −1.6 L to +2.4 L.
